# Editorial: Dysnatremias and related disorders

**DOI:** 10.3389/fmed.2024.1411974

**Published:** 2024-06-11

**Authors:** Antonios H. Tzamaloukas

**Affiliations:** ^1^Research Service, Raymond. G. Murphy Veterans Affairs Medical Center, Albuquerque, NM, United States; ^2^Department of Internal Medicine, University of New Mexico School of Medicine, Albuquerque, NM, United States

**Keywords:** dysnatremia, hyponatremia, hypernatremia, inappropriate ADH secretion, COVID-19, chronic kidney disease

The Frontiers in Medicine topic *Dysnatremias and related disorders* contains nine reports (Rohrscheib et al.; Kheetan et al.; Arzhan et al.; Do et al.; Lew et al.; Chan et al.; Vornicu et al.; Lee et al.; Kang et al.). These reports include five reviews (Rohrscheib et al.; Kheetan et al.; Arzhan et al.; Do et al.; Lew et al.), a large retrospective study (Chan et al.), and three case reports (Vornicu et al.; Lee et al.; Kang et al.). Dysnatremias, i.e., abnormally high or low values of serum sodium concentration (*[Na]*_*S*_), constitute major medical conditions. Hyponatremia is the most common electrolyte abnormality (1). Both hyponatremia and hypernatremia are associated with morbidity and mortality (2). The nine reports in this Frontiers topic address currently active issues in hyponatremia and hypernatremia.

The pathophysiology and principles of treatment constitute the central issues which must be addressed in every case with dysnatremia. Rohrscheib et al. reviewed the newer developments in this topic in relation to the key report by Edelman et al. (3). The central message from the Edelman report is a formula indicating that sodium concentration in plasma water (*[Na]*_*PW*_) is determined by exchangeable body sodium, exchangeable body potassium, and body water. According to the simpler Rose formula (4), which was derived from the Edelman formula, the only way possible for changing *[Na]*_*S*_ is to change the fraction (total body sodium + total body potassium)/body water. Rohrscheib et al. reviewed the applications of the Edelman formula in experimental and clinical studies, its role in developing formulas computing the volume of saline required for correction of dysnatremias, and newer findings providing challenges to the Edelman and Rose concept of determinants of *[Na]*_*S*_. These challenges include the following: (a) Exchanges between sodium osmotically active in body fluids and osmotically inactive sodium stored in polyanionic proteoglycans in various tissues. Such exchanges have been demonstrated in both experimental and clinical studies during conditions causing changing *[Na]*_*S*_. (b) Hydrogen binding of water to intracellular and extracellular proteins producing an exclusion zone around these proteins. Hydrogen binding potentially decreases the volume of water available for ionic solvation. And (c) genetic influences on the regulation of *[Na]*_*PW*_. The qualitative and quantitative contributions of these newer challenges to the concept expressed by Edelman during development or correction of dysnatremias will require intensive studying (Rohrscheib et al.).

Kheetan et al. reviewed the following issues encountered in hyponatremia: (a) The pathophysiological mechanisms involved in reversing the swelling of brain cells, which is the source of the clinical manifestations of hyponatremia, in acute and chronic hyponatremia. (b) The changes in various osmolytes during and following the development of hyponatremia. These changes define acute and chronic hyponatremia. Hyponatremia lasting longer than 48 h is chronic. The changes in brain organic osmolytes during development of hyponatremia determine the distinction between acute and chronic hyponatremia. (c) The influence of patient gender on the severity of hyponatremia. (d) The adverse effects of rapid correction of chronic hyponatremia on brain astrocytes, which may lead to myelinolysis. And (e) the methods for treating hyponatremia. They concluded that distinction between acute hyponatremia, which requires rapid correction, and chronic hyponatremia, which should be corrected slowly, is critical.

The conclusion of the Keetan report that distinction between acute and chronic hyponatremia should determine the rate of correction of *[Na]*_*S*_ addresses the risk of osmotic myelinolysis during treatment, a topic debated in literature recently. Rapidity of correction of hyponatremia (e.g., >8 mmol/L per 24-h) has traditionally been thought to be among the conditions predisposing to myelinolysis. Guidelines for treatment of hyponatremia contain rates of correction of *[Na]*_*S*_. However, the concept of association of myelinolysis with rapid correction of hyponatremia was recently challenged by one large study, in which only five among 3,632 patients with rapid correction of hyponatremia and seven among 19,226 patients with no rapid correction developed osmotic myelinolysis (5). Also, in a second large study correction rate of hyponatremia > 10 mmol/L per 24 h was associated with lower in-hospital mortality than a rate between 6 and 10 mmol/L per 24 h and osmotic myelinolysis was observed in five patients with rate of correction ≤ 8 mmol/L per 24 h and two patients with rate of correction > 8 mmol/L per 24 h (6). An editorial commenting on the first study called for reevaluation of the guidelines for the rate of correction of hyponatremia (7), while a review supported these guidelines (8). The report of Kheetan et al. provides a justification for why duration of hyponatremia should be considered in deciding the rate of correction of *[Na]*_*S*_. Future studies of rate of correction of hyponatremia should address the distinction between the two categories of hyponatremia.

Arzhan et al. reviewed the issues associated with dysnatremias developing in patients with chronic kidney disease (CKD). The regulation of body water, sodium, and potassium, which were identified as the determinants of *[Na]*_*PW*_ in the Edelman report (3), constitutes a major function of the kidneys. The report of Arzhan et al. reviewed the following: (a) The anatomical and functional changes that can predispose to dysnatremias in kidney failing states. (b) The incidence and prevalence of dysnatremias in patients with chronic kidney disease (CKD) treated medically or by dialysis. (c) The clinical outcomes of dysnatremias in CKD patients. (d) The conditions which increase the risk of dysnatremias. And (e) the methods of treatment and prevention of dysnatremias in this patient population. According to Arzhan et al., the areas requiring further research include the rate of correction of dysnatremias in azotemic subjects, the best dialytic method for correcting dysnatremias, and the effects of treatment on patient outcomes.

Do et al. reviewed dysnatremias secondary to gastrointestinal disorders. Large volumes of fluid containing sodium and potassium salts are ingested and secreted into the gastrointestinal tract. Gastrointestinal secretions, including gastric fluid, bile, pancreatic secretions, and small and large intestinal secretions contain various concentrations of sodium and potassium (9) and when lost externally cause dysnatremias. The report of Do et al. reviewed the following: (a) The physiological mechanisms regulating fluid and monovalent cation transfers in the gastrointestinal track. (b) The pathogenesis of hyponatremia or hypernatremia secondary to vomiting or various types of diarrhea including osmotic diarrhea, secretory diarrhea, and congenital chloride diarrhea. And (c) the treatment of dysnatremias from gastrointestinal disorders. This report stressed the association between gastrointestinal and renal functions in both the pathophysiology of water and electrolyte disorders.

Lew et al. reviewed the topic of sodium intake in patients on dialysis. High salt intake has recognized adverse effects in all populations. The adverse effects of high dietary salt content are pronounced in patients with CKD, especially those on dialysis. The report of Lew et al. addressed the following topics: (a) The clinical consequences of hypervolemia secondary to excessive salt intake in dialysis patients. (b) The recommendations for sodium intake in these patients. (c) The reported beneficial effects of prescribed strict volume control by sodium intake restriction in the dialysis populations of Tassin in France and Enge in Turkey. (d) The sodium content in food items and drug preparations. (e) The effects of high or low sodium intake on the body of patients with CKD. (f) The sociological and psychological background of sodium restriction. And (g) the sources of sodium gain and removal in various dialytic methods. The report of Lew et al. also stressed areas which need further study, such as the long term effects of methods minimizing sodium gain during the newer dialysis methods, e.g., hemodiafiltration.

Chan et al. analyzed clinical associations of hyponatremia in a large cohort of patients suffering from COVID-19. High frequencies of dysnatremias, with hyponatremia much more frequent, have been reported in patients suffering from COVID-19 (10, 11). In these patients, hyponatremia may be caused by several mechanisms and is associated with severe course, comorbidities, and mortality. In the study of Chan et al., 27.2% of the patients had hyponatremia at presentation with COVID-19. Hyponatremia was statistically associated with a large list of demographic variables, comorbidities, laboratory results, and adverse outcomes including mortality, prolonged hospitalization, and admission to intensive care units. Importantly, patients infected with the Omicron BA.2 COVID-19 variant had a higher frequency of hyponatremia, were older and had more comorbidities than patients infected with other COVID-19 strains. The report of Chan et al. underlines the need to analyze the risks for adverse events in patient populations suffering from different COVID-19 variants.

Vornicu et al. reported a case of symptomatic hyponatremia and development of Mallory-Weiss syndrome following desmopressin administration for a kidney biopsy in a 60-year old man with a liver transplant and elevated serum creatinine. These authors reviewed the literature on this topic. Desmopressin injection has been used to prevent excessive bleeding from percutaneous kidney biopsy sites (12). Water retention with development of hyponatremia constitutes the main risk of this application. The report of Vornicu et al. concludes that frequent *[Na]*_*S*_measurements and restriction of water intake should accompany desmopressin administration.

Lee et al. reported two cases of syndrome of inappropriate diuretic hormone secretion (SIADH) with exceptionally high urinary sodium concentrations (*[Na]*_*U*_) and reviewed the differential diagnosis and treatment of hyponatremia with high *[Na]*_*U*_. SIADH is a major cause of hyponatremia (13). A high *[Na]*_*U*_ accompanying the water retention and contributing to hyponatremia was documented by pitressin infusion in volunteers (14) and was subsequently observed in the first report of SIADH (15). Cerebral salt wasting (CSW) is another condition causing high *[Na]*_*U*_ and hyponatremia. The differentiation between SIADH and CSW encounters difficulties. The first case of the report of Lee et al. illustrates these difficulties. This was the case of a 40 year old man who developed severe hyponatremia and treatment difficulties after traumatic brain injury. The key difference between SIADH and CHF is the status of extracellular volume. In a typical case of SIADH the hyponatremia is euvolemic (13), while the hyponatremia in CSW is hypovolemic. The difficulty in differentiation of the two conditions is rooted in the following issues: the clinical diagnosis of hypovolemia is often very difficult, plasma ADH levels are high in both CSW, secondary to hypovolemia, and SIADH, and plasma levels of natriuretic peptides may be low, normal, or high in CSW (16). The fractional excretions of urate and phosphate after correction of the hyponatremia and measurement of the level of a natriuretic haptoglobin-related protein may clarify the distinction between SIADH and CSW (16, 17). However, the proper steps in differentiating between SIADH and CSW are currently disputed (17–19). Further research in this area is required.

Kang et al. reported a case of severe hypernatremia developing in a patient ingesting his own urine for 5 days. Loss of body water in excess of sodium and potassium losses leads to hypernatremia (4). Kang et al. proposed that one mechanism that could contribute to the development of hypernatremia in their patient was osmotic diuresis secondary to high loads of urea in the ingested urine. Indeed, serum urea and serum creatinine were elevated on admission in this patient and were normalized after a few days of fluid and electrolyte infusion. Osmotic diuresis causes hypernatremia because of excessive water loss documented by a sum of urinary sodium plus potassium concentrations lower than a normal *[Na]*_*S*_, even though urine osmolality is higher than serum osmolality (20). The case reported by Kang et al. supports this mechanism of hypernatremia. During treatment, the urine osmolality of their patient was persistently higher than his serum osmolality, while repeated measurements revealed urinary sodium concentrations substantially below the normal range of *[Na]*_*S*_. Urinary potassium was not measured.

The nine reports in this Frontiers topic provided information which the authors and editors hope can help those managing patients with dysnatremias. Areas needing research were also identified in several reports on this topic and are shown in this editorial.

## Author contributions

AT: Conceptualization, Methodology, Writing – original draft.

## In memoriam

This editorial is dedicated to the memory of Dr. Todd S. Ing, MD. Dr. Ing, who passed away relatively recently, conceived this Frontiers topic, edited one of its manuscripts, and made important contributions to three of its manuscripts.
